# The Wetting Deformation Model of Rockfill and Its Two Methods for Simulating Rockfill Dam Collapse Settlement

**DOI:** 10.1155/2023/5593636

**Published:** 2023-10-12

**Authors:** Litan Pan, Zhidong Fan, Daquan Wang, Xiongxiong Zhou

**Affiliations:** ^1^Huadian Electric Power Research Institute Company Limited, Hangzhou, Zhejiang 310000, China; ^2^Key Laboratory of Agricultural Soil and Water Engineering in Arid and Semiarid Areas of Ministry of Education, Northwest A & F University, Yangling, Shaanxi 712100, China; ^3^College of Water Resources and Architectural Engineering, Northwest A & F University, Yangling, Shaanxi 712100, China

## Abstract

The wetting deformation of the upstream dam shell material during the impoundment of the core wall rockfill dam seriously affects the safety of the dam. Based on the proposed *E*^*w*^ − *ν*^w^ wetting model, this paper proposes its corresponding two methods to simulate the collapse settlement of the rockfill dam: the initial strain method and the initial stress method. By simulating the collapse settlement of the Guanyinyan core wall rockfill dam, it is found that the simulated result using the initial stress method is in good agreement with the field monitoring data, while the displacement simulated using the initial strain method is larger. The distribution of displacement contours simulated using the initial strain method is obviously inconsistent in the area where the wetting deformation occurs, and the simulation results of the initial stress method are more reasonable. With the rise in the water level, the wetting deformation of the upstream dam shell material causes the tensile stress zone at the top of the dam. Therefore, the wetting deformation is the direct cause of the crack at the top of the dam, and the initial stress method should be preferred in the simulation of the wetting deformation of rockfill materials.

## 1. Introduction

Wetting deformation of rockfill refers to the deformation of rockfill caused by soaking in water under a certain stress state. The reason is that the particles are soaked and softened and the particles are contacted and lubricated, which triggers the imbalance of the force on the particles, causing the particles to break, rearrange, adjust, and gradually restore the balance so that the stress in the rockfill is redistributed and deformed. The collapse deformation caused by the upstream rockfill wetting deformation during the impoundment of the core wall rockfill dam often affects the safe operation of the dam. In light cases, it will produce collapse cracks at the top of the dam. In severe cases, it will cause deep cracks in important parts such as dam abutments and even form leakage channels, threatening the safety of the dam [1, 2]. During the impoundment of the Xiaolangdi inclined core wall dam [3], Pubugou high core wall rockfill dam [4], and Guanyinyan core wall rockfill dam [5], the differential settlement of the dam crest is caused by the wetting deformation of the upstream dam shell material and longitudinal cracks are generated at the dam crest. Therefore, it is very important to study the wetting deformation of rockfill and its effective simulation in rockfill dams.

The study of wetting deformation of an earth-rock dam is usually divided into two processes. First, a laboratory triaxial wetting test is carried out to judge the wetting deformation mode of the earth-rock dam, establish the wetting deformation model of the earth-rock dam, and attain the parameters of the wetting deformation model of the earth-rock dam. Then, the established wetting deformation model is applied to the finite element simulation calculation of the earth-rock dam, and the deformation caused by the wetting of the rockfill material is simulated to analyze whether the dam body will produce cracks and then judge the safety of the actual project.

The oedometer test and the triaxial test are generally used to study the wetting deformation characteristics of rockfill materials in laboratory experiments. There are generally two kinds of wetting test methods for rockfill materials, namely, the double-line method and the single-line method. The double-line method is used to carry out the triaxial test of the natural water content sample and the saturated sample, respectively. The wetting deformation of the double-line method is calculated as the deformation difference between the natural water content sample and the saturated sample under the same stress state. The single-line method is used to compress the natural water content sample to different deviatoric stress levels and then slowly soak and saturate under the premise of keeping the deviatoric stress unchanged. The wetting deformation of the single-line method is the strain corresponding to the change of the sample from the natural moisture content to the saturation state. Bao and Qu [6] proposed that it is unreasonable to replace the strength parameters, deformation parameters, and stress-strain relationship of rockfill materials from natural moisture content or dry state to saturated state with the strength parameters, deformation parameters, and stress-strain relationship of generally saturated rockfill materials. Chen et al. [7] also proposed that the development of stress-strain relationship in a single-line triaxial wetting test is more in line with the actual situation of rockfill wetting. Based on the above premise, the single-line wetting test is preferred in the study of wetting deformation of rockfill materials.

Many researchers have proposed simulation methods for the numerical simulation of wetting deformation. Based on the nonlinear elastic constitutive model, Nobari and Duncan [8, 9] used the curve fitting method to calculate the wetting unbalanced stress and simulated the collapsible settlement of the Oroville Dam. The numerical simulation of the collapsible settlement of the dam was realized for the first time. Wei [10] proposed a wetting plastic model based on the double yield surface elastoplastic constitutive model and simulated the wetting deformation of Nuozhadu Dam. Bauer [11, 12] found that the degree of saturation can change the solid hardness through the elastoplastic constitutive model and calculated the stress relaxation by using the degradation model of hardness so as to simulate the wetting deformation during the impoundment of the dam by reducing the hardness of rockfill [13]. Based on the nonlinear elastic constitutive model, Chi and Zhou [14] studied the wetting strain development model of rockfill materials during the soaking process on the basis of a single-line triaxial wetting test, summarized and proposed the wetting model, and simulated the wetting deformation during the first impoundment of the dam [15].

From the experimental point of view, it is more reasonable to simulate the wetting deformation based on the initial strain method of the single-line wetting test. However, when the initial strain is applied to the dam body, the virtual equivalent node load method is generally used. In this process, the integration of element stiffness and initial strain is needed to calculate the node load. The difference between the loading modulus and unloading modulus of the rockfill material can be several times. During the impoundment of the core rockfill dam, the water load, buoyancy, and wetting deformation making the adjacent elements may be in different states of loading and unloading. The huge difference in the stiffness matrix will inevitably lead to stress singularity and deformation incompatibility in the calculation results. The initial stress method can avoid the above shortcomings of the initial strain method. However, the determination method for wetting initial stress based on the single-line wetting test is rarely studied by scholars. In addition, the generation of wetting deformation has a time effect, not instantaneous deformation, so the simulation of wetting deformation should also consider the law of strain development during the wetting process. Based on the nonlinear elastic theory, the authors used the method of modulus reduction, combined with the characteristics of the wetting process and the final wetting deformation in the single-line wetting test, and gave the *E*^*w*^ − *ν*^*w*^ wetting deformation model which can fit the test data well without using the flow rule [14, 15].

In the first part of this paper, the proposed *E*^*w*^ − *ν*^*w*^ wetting deformation model is introduced. Then, based on this model, the process of simulating rockfill dam collapse deformation using the initial strain method and initial stress method is introduced, respectively. Finally, the above method for simulating collapse deformation is applied to practical engineering, and the simulation results of the two methods are compared with the actual monitoring data.

## 2. Wetting Model

Based on the analysis of a large number of single-line wetting test results of rockfill materials, a wetting deformation model of rockfill materials is proposed in this paper. Among them, the wetting stress level and the wetting axial strain and confining pressure satisfy the formula as follows:

(1) $$\begin{array}{c} ∆\varepsilon_{a}^{w}=\frac{\left[K_{1}\left(\sigma_{3}/Pa\right)+A\right]S_{L}}{1-S_{L}}+K_{0}{\left(\frac{\sigma_{3}}{Pa}\right)}^{m}, \end{array}$$

where ∆*ε*_*a*_^*w*^ is the wetting axial strain, *σ*_3_ is the confining pressure, *P*_*a*_ is the standard atmospheric pressure, *S*_*L*_=(*σ*_1_ − *σ*_3_)/(*σ*_1_ − *σ*_3_)_*f*_ is the wetting stress level, (*σ*_1_ − *σ*_3_)_*f*_=(2*c* cos *ϕ*+2*σ*_3_sin *ϕ*)/(1 − sin *ϕ*) is the shear strength of air-dried samples, *c* and *ϕ* are the cohesion and friction angle, and *K*_1_, *A*, *K*_0_, and *m* are the parameters.

At the same time, it is found that the relationship curve between the volume strain change and the axial strain change remains a straight line during the wetting process and the curve slope decreases with the increase of the wetting stress level; that is, the ratio *k* of the wetting volume strain increment to the axial strain increment is constant and decreases with the increase of the wetting stress level.

**Figure 1:**
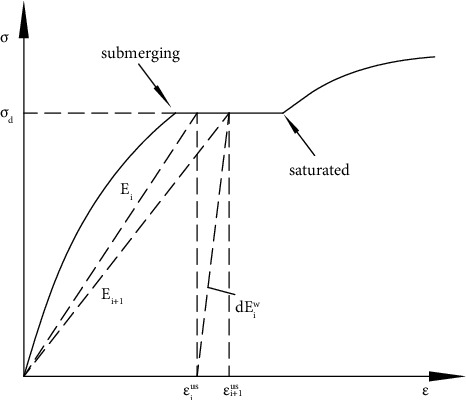
Modulus softening and incremental wetting strain during the wetting process.

When nonlinear elastic theory is used to simulate the wetting deformation of rockfill, it is considered that the wetting deformation is caused by the reduction of secant modulus. The reduction of the secant modulus of the material, i.e., the softening of the material, will cause the adjustment of the internal stress deformation relationship of the material, resulting in wetting deformation. As shown in Figure 1, the secant modulus of *i* at any time in wetting is *E*_*i*_ and the strain is *ε*_*i*_^*us*^=*σ*_*d*_/*E*_*i*_; with the development of wetting deformation, the secant modulus at the next moment becomes *E*_*i*+1_ and the strain becomes *ε*_*i*+1_^*us*^=*σ*_*d*_/*E*_*i*+1_; then, the wetting strain *dε*_*i*_^*w*^ in this period satisfies the following relationship:

(2) $$\begin{array}{c} d\varepsilon_{i}^{w}=\frac{\sigma_{d}}{dE_{i}^{w}}=\varepsilon_{i+1}^{us}-\varepsilon_{i}^{us}=\frac{\sigma_{d}}{E_{i+1}}-\frac{\sigma_{d}}{E_{i}}=\frac{E_{i}-E_{i+1}}{E_{i}E_{i+1}}\sigma_{d}, \end{array}$$

where *dE*_*i*_^*w*^ is the wetting secant modulus in this period, as shown in Figure 1, and there are the following relationships:

(3) $$\begin{array}{c} dE_{i}^{w}=\frac{E_{i}E_{i+1}}{E_{i}-E_{i+1}}. \end{array}$$

Under the triaxial stress state, the incremental wetting axial strain *dε*_*a*,*i*_^*w*^ and wetting volume strain *dε*_*v*,*i*_^*w*^ have the following expressions:

(4) $$\begin{array}{c} d\varepsilon_{a,i}^{w}=\frac{\sigma_{1}}{dE_{i}^{w}}-2\nu_{i}^{w}\frac{\sigma_{3}}{dE_{i}^{w}}=\frac{\left(\sigma_{1}-2\nu_{i}^{w}\sigma_{3}\right)}{dE_{i}^{w}}, \end{array}$$

(5) $$\begin{array}{c} d\varepsilon_{v,i}^{w}=d\varepsilon_{a,i}^{w}+2d\varepsilon_{r,i}^{w}=\frac{\left(\sigma_{1}-2\nu_{i}^{w}\sigma_{3}\right)}{dE_{i}^{w}}+\frac{\left(\left(1-\nu_{i}^{w}\right)\sigma_{3}-\nu_{i}^{w}\sigma_{1}\right)}{dE_{i}^{w}}=\frac{\left(\sigma_{1}+2\sigma_{3}\right)\left(1-2\nu_{i}^{w}\right)}{dE_{i}^{w}}, \end{array}$$

where *ν*_*i*_^*w*^ is Poisson's ratio in this section and d*ε*_*r*,*i*_^*w*^ is the radial wetting strain increment. During this period, the ratio *k*_*i*_ of wetting volume strain increment to wetting axis strain increment has the following relationship:

(6) $$\begin{array}{c} k_{i}=\frac{d\varepsilon_{v,i}^{w}}{d\varepsilon_{a,i}^{w}}=\frac{\left(\sigma_{1}+2\sigma_{3}\right)\left(1-2\nu_{i}^{w}\right)}{\left(\sigma_{1}-2\nu_{i}^{w}\sigma_{3}\right)}. \end{array}$$

The wetting Poisson's ratio in this period is as follows:

(7) $$\begin{array}{c} \nu_{i}^{w}=\frac{1+2\sigma_{3}/\sigma_{1}-k_{i}}{2+4\sigma_{3}/\sigma_{1}-2k_{i}\sigma_{3}/\sigma_{1}}. \end{array}$$

The ratio *k*_*i*_ during the wetting process is a constant; in the wetting test of the “single line method,” the stress state is constant; that is, *σ*_3_/*σ*_1_ is a constant in the wetting process. Therefore, according to formula (7), Poisson's ratio *ν*^w^ is a constant in the wetting process.

(8) $$\begin{array}{c} \nu^{w}=\nu_{0}^{w}=\nu_{1}^{w}=\ldots =\nu_{k}^{w}=\ldots . \end{array}$$

Through the study of the “single line method” test data of many scholars, it is found that the wetting Poisson's ratio has no obvious correlation with the confining pressure during wetting and satisfies the linear relationship with the wetting stress level.

(9) $$\begin{array}{c} \nu^{w}=c+dS_{L}, \end{array}$$

where *c* and *d* are the test parameters.

By combining formula (8) and accumulating the wetting axis strain increment in formula (4), the total wetting axis increment ∆*ε*_*a*_^*w*^ can be obtained.

(10) $$\begin{array}{c} ∆\varepsilon_{a}^{w}=\underset{i=0}{\sum }d\varepsilon_{a,i}^{w}=\left(\sigma_{1}-2\nu^{w}\sigma_{3}\right)\overset{n}{\underset{i=0}{\sum }}\frac{1}{{dE}_{i}^{w}}, \end{array}$$

where *n* is the number of accumulated periods.

It can be seen from the above that the cumulative wetting axis increment is essentially the reciprocal of the cumulative wetting secant modulus. Combined with equation (3), the following relationship can be obtained:

(11) $$\begin{array}{c} \overset{n}{\underset{i=0}{\sum }}\frac{1}{{dE}_{i}^{w}}=\overset{n}{\underset{i=0}{\sum }}\frac{E_{i}-E_{i+1}}{E_{i}E_{i+1}}=\frac{E_{0}-E_{1}}{E_{0}E_{1}}+\ldots +\frac{E_{i}-E_{i+1}}{E_{i}E_{i+1}}+\ldots \frac{E_{n-1}-E_{n}}{E_{n-1}E_{n}}. \end{array}$$

**Figure 2:**
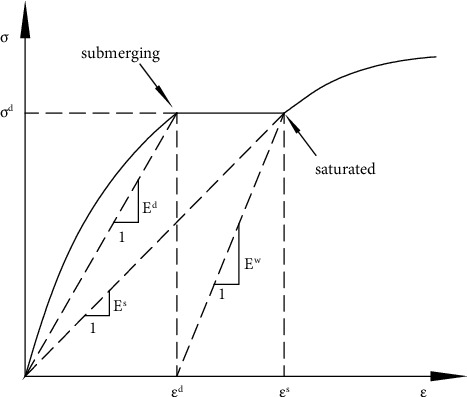
Modulus changes before and after wetting.

In the formula, *E*_0_ is the initial secant model of the sample, that is, the secant modulus *E*^*d*^ before wetting, and *E*_*n*_ is the final secant model of the sample, that is, the secant modulus *E*^*s*^ after wetting, as shown in Figure 2. Then, formula (11) can be changed into the following formula:

(12) $$\begin{array}{c} \underset{i=0}{\sum }\frac{1}{{dE}_{i}^{w}}=\frac{E^{d}-E_{1}}{E^{d}E_{1}}+\frac{E_{1}-E_{2}}{E_{1}E_{2}}\ldots +\frac{E_{i}-E_{i+1}}{E_{i}E_{i+1}}+\ldots \frac{E_{n-1}-E^{s}}{E_{n-1}E^{s}}\\ =\frac{E^{d}-E_{2}}{E^{d}E_{2}}+\ldots +\frac{E_{i}-E_{i+1}}{E_{i}E_{i+1}}+\ldots \frac{E_{n-1}-E^{s}}{E_{n-1}E^{s}}=\frac{E^{d}-E^{s}}{E^{d}E^{s}}=\frac{1}{E^{w}}, \end{array}$$

where *E*^*w*^ is the wetting secant modulus.

By taking formula (12) into formula (10), the following formula can be obtained:

(13) $$\begin{array}{c} ∆\varepsilon_{a}^{w}=\frac{\sigma_{1}-2\nu^{w}\sigma_{3}}{E^{w}}. \end{array}$$

Combining it with equation (1), the calculation method for the wetting secant modulus can be obtained.

(14) $$\begin{array}{c} E^{w}=\frac{\sigma_{1}-2\nu^{w}\sigma_{3}}{\left[K_{1}\left(\sigma_{3}/P_{a}\right)+A\right]S_{L}/1-S_{L}+K_{0}{\left(\sigma_{3}/P_{a}\right)}^{m_{0}}}. \end{array}$$

Therefore, the method of calculating the wetting secant modulus (formula (14)) and the wetting Poisson's ratio (formula (9)) in the *E*^*w*^ − *ν*^*w*^ wetting model was determined.

## 3. Two Methods for Simulating Wetting Deformation

The *E*^*w*^ − *ν*^*w*^ wetting model is based on the nonlinear elastic theory to study the wetting deformation of rockfill materials, combined with the variation law of wetting deformation experimental data and the mechanical conditions. According to the generalized Hooke's law, the wetting strain can be directly calculated from the wetting secant modulus and the wetting Poisson's ratio and the wetting deformation can be simulated by the initial strain method. The reduction of stress can also be calculated by calculating the reduction of the secant modulus in the wetting process, which is used as the initial stress to simulate the wetting deformation.

### 3.1. Initial Strain Method

First, according to the generalized Hooke's law, the wetting strain is calculated from the wetting secant modulus *E*^*w*^ and the wetting Poisson's ratio *ν*^*w*^.

(15) $$\begin{array}{c} \left\{\begin{array}{c} ∆\varepsilon_{x}^{w}\\ ∆\varepsilon_{y}^{w}\\ ∆\varepsilon_{z}^{w}\\ ∆\gamma_{xy}^{w}\\ ∆\gamma_{yz}^{w}\\ ∆\gamma_{zx}^{w} \end{array}\right\}=\frac{1}{E_{w}}\left[\begin{array}{cccccc} 1 & -\nu_{S} & -\nu_{S} & 0 & 0 & 0\\ -\nu_{S} & 1 & -\nu_{S} & 0 & 0 & 0\\ -\nu_{S} & -\nu_{S} & 1 & 0 & 0 & 0\\ 0 & 0 & 0 & 2\left(1+\nu_{S}\right) & 0 & 0\\ 0 & 0 & 0 & 0 & 2\left(1+\nu_{S}\right) & 0\\ 0 & 0 & 0 & 0 & 0 & 2\left(1+\nu_{S}\right) \end{array}\right]\left\{\begin{array}{c} \sigma_{x}\\ \sigma_{y}\\ \sigma_{z}\\ \tau_{xy}\\ \tau_{yz}\\ \tau_{zx} \end{array}\right\}. \end{array}$$

Then, as the initial strain, it is transformed into a virtual equivalent node load:

(16) $$\begin{array}{c} {\left\{f\right\}}^{s}=\sum {∭}_{V}{\left[B\right]}^{T}\left[D\right]\left\{∆\varepsilon^{w}\right\}\text{dV}. \end{array}$$

The virtual equivalent node load is applied to the wetting element to consider the wetting deformation when the rockfill dam is under impoundment.

Finally, the stress corresponding to the corresponding virtual equivalent node load needs to be deducted from the total stress.

(17) $$\begin{array}{c} {\left\{∆\sigma^{w}\right\}}^{s}=\left[D\right]\left\{∆\varepsilon^{w}\right\}. \end{array}$$

**Figure 3:**
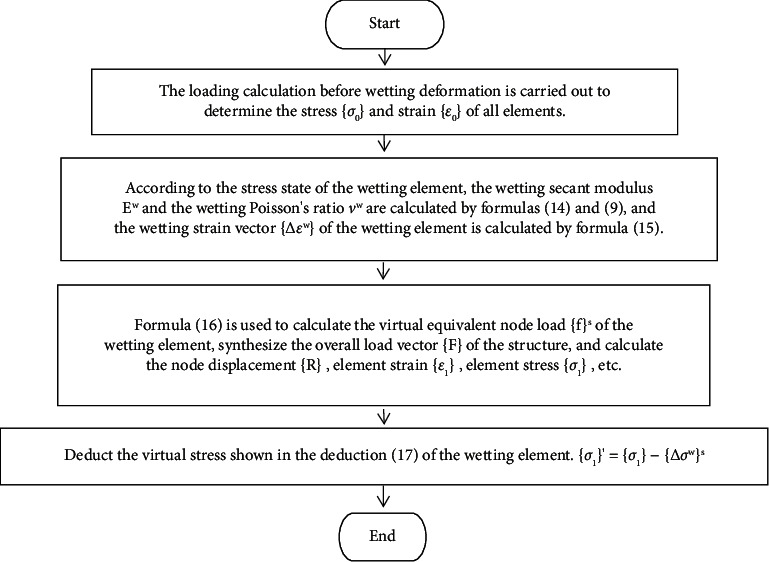
“Wetting initial strain method” simulating the wetting deformation process.

The calculation process is shown in Figure 3.

Compared with other wetting deformation models, the *E*^*w*^ − *ν*^*w*^ wetting model is used to calculate the wetting strain, which can avoid using the flow rule to distribute the wetting volumetric strain and wetting shear strain in all directions.

### 3.2. Initial Stress Method

The *E*^*w*^ − *ν*^*w*^ wetting deformation model is derived based on the nonlinear elastic theory, using the modulus softening method, combined with the wetting deformation law. The essence is to regard the wetting deformation as elastic deformation, which is theoretically in conflict with the initial strain method which regards the wetting deformation as plastic deformation. Therefore, the author will calculate the stress reduction in the wetting process by modulus softening and use the reduced stress as the initial stress to simulate the wetting deformation.

**Figure 4:**
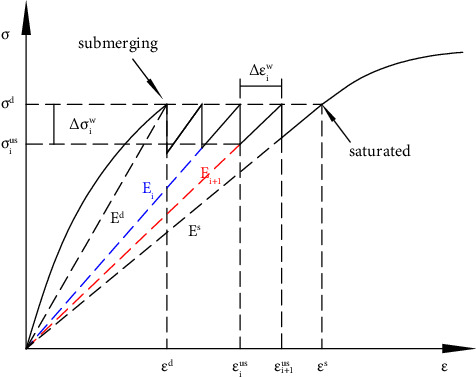
Change process of modulus, stress, and strain during the wetting process.

As shown in Figure 4, the process of rockfill wetting can be regarded as the repeated process of specimen modulus softening, stress reduction, strain increase, and equilibrium restoration. This process continues until the sample reaches a stable saturation state; that is, the modulus softens from the state *E*^*d*^ before wetting to the state *E*^*s*^ in the presence of water. In a small period, the secant modulus of the sample is reduced from *E*_*i*_ to *E*_*i*+1_, the stress is reduced from *σ*^*d*^ to *σ*_*i*_^*us*^, the strain is increased from *ε*_*i*_^*us*^ to *ε*_*i*+1_^*us*^, and the balance is restored. The wetting stress ∆*σ*_*i*_^*w*^ and wetting strain ∆*ε*_*i*_^*w*^ in this process satisfy the following relationship:

(18) $$\begin{array}{c} ∆\sigma_{i}^{w}=\sigma^{d}-\sigma_{i}^{us}=\sigma^{d}-\sigma^{d}\frac{E_{i+1}}{E_{i}}=\sigma^{d}\frac{E_{i}-E_{i+1}}{E_{i}},\\ ∆\varepsilon_{i}^{w}=\varepsilon_{i+1}^{us}-\varepsilon_{i}^{us}=\frac{\sigma^{d}}{E_{i+1}}-\frac{\sigma^{d}}{E_{i}}=\frac{∆\sigma_{i}^{w}}{E_{i+1}}. \end{array}$$

In the three-dimensional stress space, the wetting stress {∆*σ*_*i*_^*w*^} caused by the decrease of modulus from *E*_*i*_ to *E*_*i*+1_ during wetting is shown as follows:

(19) $$\begin{array}{c} \left\{∆\sigma_{i}^{w}\right\}=\left\{\sigma^{d}\right\}-\left\{\sigma_{i}^{us}\right\}=\left\{\sigma^{d}\right\}-{\left[D_{i}\right]}^{-1}\left[D_{i+1}\right]\left\{\sigma^{d}\right\}={\left[D_{i}\right]}^{-1}\left(\left[D_{i}\right]-\left[D_{i+1}\right]\right)\left\{\sigma^{d}\right\}, \end{array}$$

where {*σ*^*d*^} and {*σ*_*i*_^*us*^} are the stress tensors before and after the modulus decreases and [*D*_*i*_] and [*D*_*i*+1_] are the elastic matrices before and after the modulus decreases. It can be seen from Section 3.2.3 that the wetting Poisson's ratio is constant during the wetting process, so there is the following formula:

(20) $$\begin{array}{c} {\left[D_{i}\right]}^{-1}\left(\left[D_{i}\right]-\left[D_{i+1}\right]\right)=\frac{E_{i}-E_{i+1}}{E_{i}}\left[I\right], \end{array}$$

where [*I*] is the unit matrix. Bring formula (20) into formula (19), and the following formula can be obtained.

(21) $$\begin{array}{c} \left\{∆\sigma_{i}^{w}\right\}=\left\{\sigma^{d}\right\}\frac{E_{i}-E_{i+1}}{E_{i}}. \end{array}$$

The wetting strain {∆*ε*_*i*_^*w*^} produced by this wetting stress is as follows:

(22) $$\begin{array}{c} \left\{∆\varepsilon_{i}^{w}\right\}={\left[D_{i+1}\right]}^{-1}\left\{∆\sigma_{i}^{w}\right\}. \end{array}$$

According to Figure 2, the secant modulus difference ∆*E* before and after wetting, that is, the total amount of modulus reduction during wetting, satisfies the following relationship:

(23) $$\begin{array}{c} ∆E=E^{d}-E^{s}=\frac{{\left(E^{d}\right)}^{2}}{E^{d}+E^{w}}. \end{array}$$

The secant modulus before wetting was calculated using the secant modulus calculation method in the Duncan–Zhang model.

(24) $$\begin{array}{c} E^{d}=KP_{a}{\left(\frac{\sigma_{3}}{P_{a}}\right)}^{n}\left(1-R_{f}S\right), \end{array}$$

where *K*, *n*, *R*_*f*_, and *s* are the modulus number, modulus exponent, failure ratio, and stress level, respectively.

In the simulation of the wetting deformation of the dam, the total modulus reduction ∆*E* is calculated first; then, gradually soften the modulus (similar to Figure 4, *n*-step softening modulus), calculate the stress reduction, that is, the wetting stress {∆*σ*_*i*_^*w*^} caused by the modulus reduction, and perform stress reduction on the corresponding wetting element; finally, the equivalent node load method is used to calculate the wetting deformation, and the calculation formula of element equivalent node load {*f*} is as follows:

(25) $$\begin{array}{c} \left\{f\right\}={∭}_{v}{\left[B\right]}^{T}\left\{∆\sigma_{i}^{w}\right\}dv. \end{array}$$

**Figure 5:**
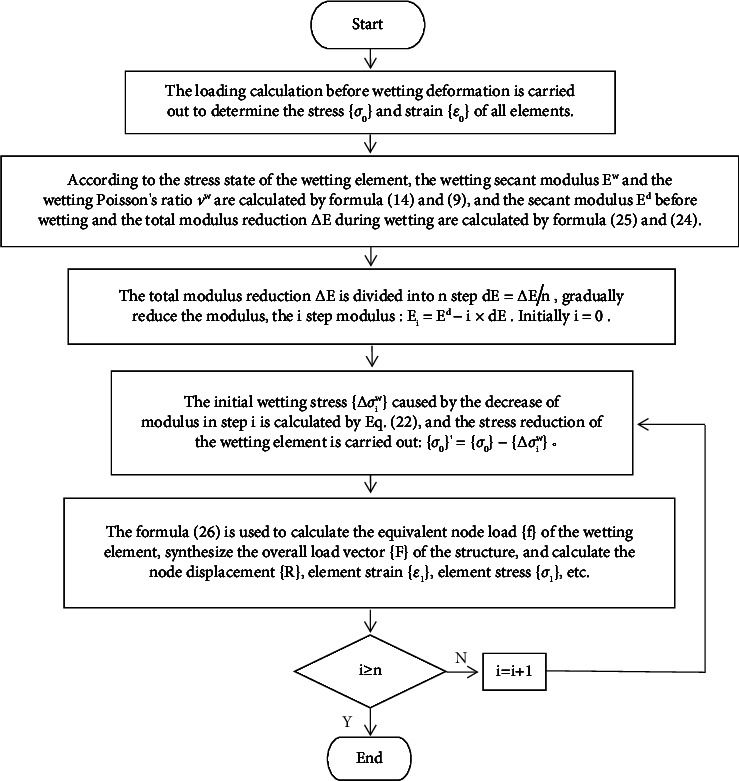
“Wetting initial stress method” simulating the wetting deformation process.

The simulation process is shown in Figure 5.

## 4. Simulation of Collapse Settlement of Guanyinyan Rockfill Dam

The calculation of static deformation of rockfill dams is divided into three parts: transient deformation due to the first filling during the construction period, collapsibility settlement during the storage period, and creep deformation during the operation period. There are corresponding models to simulate the deformation of each part, namely, the constitutive model, wetting model, and creep model. This paper mainly simulates the filling and impoundment process of a Guanyinyan core rockfill dam. According to the Duncan Zhang EB constitutive model (nonlinear elastic model), the deformation of the dam under gravity load during the construction period and water load during the impoundment period is calculated. The seven-parameter creep model is used to simulate the creep deformation of the dam. The *E*^*w*^ − *ν*^*w*^ wetting deformation model and the two wetting deformation simulation methods introduced in Section 2 are used. The wetting deformation of the dam and the difference between the results of two different wetting deformation simulation methods are analyzed.

### 4.1. Project Profile

**Figure 6:**
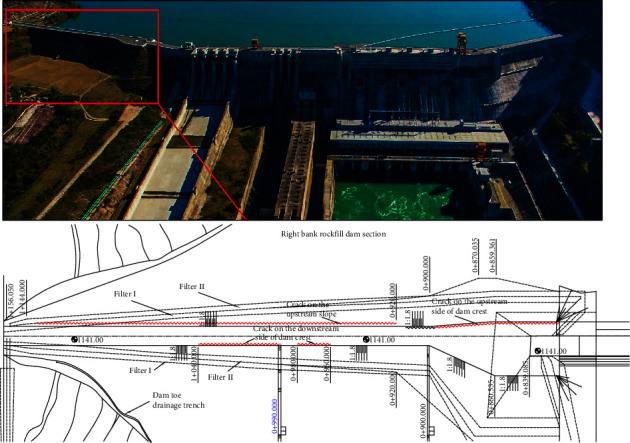
Guanyinyan Dam and the location of cracks on the top of the right bank rockfill dam.

**Figure 7:**
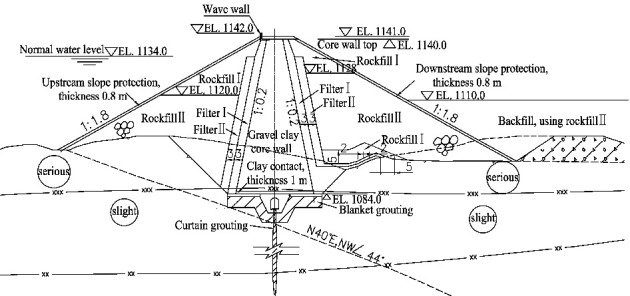
Typical cross section of Guanyinyan Dam (EL: elevation).

The Guanyinyan Hydropower Station, located in the middle reaches of Jinsha River in Yunnan Province, China, is the last station of eight hydropower stations planned in the middle reaches of Jinsha River. The project has the functions of flood control, power generation, reservoir navigation, tourism, and so on. As shown in Figure 6, the Guanyinyan Dam is composed of a clay core rockfill dam and a gravity dam on the right bank, and the two are connected by a 75 m high insertion joint. The Guanyinyan Dam is a partition rockfill dam. The maximum dam height is 75 m, the maximum top elevation is 1141 m, the top elevation of the core wall is 1140.0 m, the slope ratio of the upper and lower reaches of the core wall is 1 : 0.2, the bottom elevation of the dam is 1185.0 m, and the slope ratio of the upper and lower reaches of the dam is 1 : 1.8. The typical cross section of the Guanyinyan Dam is shown in Figure 7. This section is mainly composed of six parts: clay core wall, filter layer I, filter layer II, rockfill body I, rockfill body II, and backfill materials [16, 17].

The construction of the rockfill area began in August 2012, and the construction of the core wall began in March 2013. On April 15, 2014, the core wall was filled to a design elevation of 1140 m. On 23^rd^ October 2014, the reservoir ceased to hold water. When the water level of the reservoir reached 1110 m on November 20, 2014, cracks appeared at the top of the dam connecting the concrete dam and the core wall rockfill dam. The cracks are mainly distributed at the joint of the core wall and its upstream side. With the development of cracks, cracks also appear on the top of the core wall rockfill dam. On November 26, 2014, when the reservoir water level reached 1117 m, six cracks appeared in the connection area between the concrete dam and the core wall dam. The longest crack is 25 m, the maximum crack width is 5 cm, and the depth is about 5.5 m. There are four longitudinal cracks near the dam crest on the upstream and downstream sides of the core wall dam, and the longest crack on the upstream side is 127 m, as shown in Figure 6. The wetting deformation of upstream rockfill is the key factor leading to dam cracking. Therefore, this paper analyzes and studies the wetting deformation and cracks of the Guanyinyan Dam [18, 19].

### 4.2. Parameters of the Constitutive Model, Creep Model, and Wetting Deformation Model

In the Duncan–Chang EB constitutive model, the nonlinear stress-strain relationship is expressed by hyperbola. The instantaneous slope of the curve is tangent modulus *E*_*t*_, and the relationship is expressed as follows:

(26) $$\begin{array}{c} E_{t}=K\cdot P_{a}{\left(\frac{\sigma_{3}}{P_{a}}\right)}^{n}{\left[1-R_{f}\left(\sigma_{1}-\sigma_{3}\right)\frac{\left(1-\sin\;\phi \right)}{2c\cdot \cos\;\phi +2\sigma_{3}\cdot \sin\;\phi }\right]}^{2}. \end{array}$$

The bulk modulus can be expressed as follows:

(27) $$\begin{array}{c} B=K_{b}\cdot P_{a}{\left(\frac{\sigma_{3}}{P_{a}}\right)}^{m}, \end{array}$$

where *K*_*b*_ is the bulk modulus number and *m* is the bulk modulus exponent.

Almost all the Mohr–Coulomb envelopes of the soil in contact have varying degrees of bending, and the wider the confining pressure range, the greater the bending degree, especially for noncohesive soil such as sand, gravel, and rockfill. For example, near the middle bottom of the dam, where the rockfill body is subjected to excessive pressure, the friction angle of the rockfill in the middle bottom of the dam may be several degrees smaller than that of the rockfill near the surface of the slope. This change can be described by the following equation:

(28) $$\begin{array}{c} \phi =\phi_{0}-∆\phi \;\log\left(\frac{\sigma_{3}}{P_{a}}\right), \end{array}$$

where *ϕ*_0_ is the value of *ϕ* for *σ*_3_=*P*_*a*_ and ∆*ϕ* is the reduction in *ϕ* for a 10-fold increase in *σ*_3_. There are seven parameters (i.e., *c*, *ϕ* (or *ϕ*_0_, ∆*ϕ*), *R*_*f*_, *K*, *n*, *K*_*b*_, and *m*), which can be evaluated by using a group of conventional triaxial tests.  Table 1 shows the model parameters which are determined according to the report of the experimental study on material late deformation of the core wall rockfill dam in Guanyinyan Hydropower Station [20].

The creep deformation of the dam is simulated based on the seven parameters' creep model, in which the Merchant equation is used to describe the creep curve,

(29) $$\begin{array}{c} \varepsilon \left(t\right)=\varepsilon_{i}+\varepsilon_{f}\left(1-e^{-\omega t}\right), \end{array}$$

where *ε*(*t*) is the creep strain developed at time *t*, *ε*_*i*_ and *ε*_*f*_ are the initial and permanent creep strains, respectively, *e* is the natural index, and *ω* is a parameter that represents the initial relative deformation rate (or creep strain during the first day). Fang [21] gave the improved calculation formulas of permanent volumetric creep strain *ε*_*vf*_ and permanent shear creep strain *γ*_*f*_, as is shown in the following equation:

(30) $$\begin{array}{c} \left\{\begin{array}{c} \varepsilon_{vf}=b_{1}{\left(\frac{\sigma_{3}}{P_{a}}\right)}^{m_{1}}+c_{1}{\left(\frac{q}{P_{a}}\right)}^{m_{2}},\\ \gamma_{f}=d_{1}{\left(\frac{S}{1-S}\right)}^{m_{3}}, \end{array}\right. \end{array}$$

where *b*_1_, *c*_1_, *d*_1_, *m*_1_, *m*_2_, and *m*_3_ are the parameters.  Table 2 lists the back analysis creep model parameters of the main dam materials of the Guanyinyan Dam, which are determined by referring to the research results of Jia et al. [5].

The *E*^*w*^ − *ν*^*w*^ wetting deformation model and the simulation method of wetting deformation are shown in Section 2. The parameters involved are taken from the late deformation test report of the core rockfill dam material of Guanyinyan Hydropower Station [20], as shown in Table 3.

### 4.3. Simulation Process Introduction

Based on the analysis and research of the Guanyinyan Dam structure and monitoring data, it was found that the 0 + 990 m section of the dam (as shown in Figure 6) is close to the plane strain state. Therefore, this paper simulates the filling and water storage processes of this section. Based on the C/C++ finite element static analysis program of earth-rock dam developed by the authors, this section adds a module to simulate wetting deformation by using the *E*^*w*^ − *ν*^*w*^ wetting deformation model and its two simulation methods, initial strain method and initial stress method, and applies them to the simulation of collapse settlement during dam impoundment.

**Figure 8:**
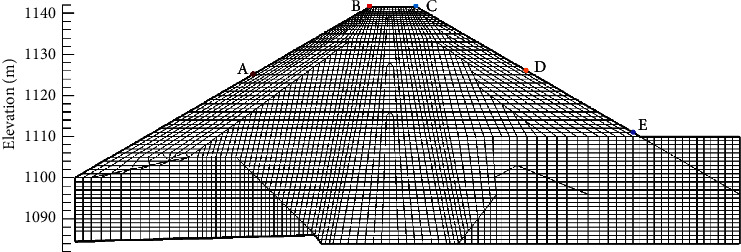
Finite element mesh model of dam 0 + 990.0 m section.

**Figure 9:**
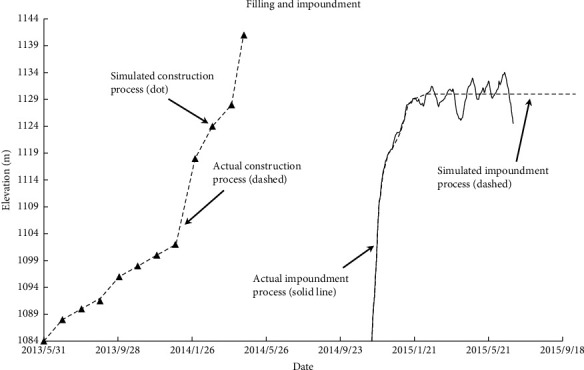
Process of filling and impoundment.

The finite element mesh model established for calculation in this paper is shown in Figure 8. The finite element model consists of 4406 elements and 4439 nodes. In the vertical direction, the mesh size is 1.0 m below the elevation of 1137.0 m and 0.5 m above the elevation of 1137.0 m. According to the actual water storage process of the dam, the simulated water storage load step is added and the simulated water level of the reservoir remains unchanged after reaching the normal water level. There are 107 fill load steps and 45 water storage load steps in the simulation process. Considering the creep deformation of dam materials in the process of filling and water storage, 18 creep load steps are inserted into the filling load step during the simulation according to the actual construction process to consider the creep deformation caused by filling. According to the actual change process of the reservoir water level, 40 creep load steps are inserted into the water storage load step to consider the creep deformation caused by water storage. The actual filling, water storage, and simulation process of the dam are shown in Figure 9. In the process of filling the dam, water pressure is applied upstream of the core wall. Seepage in the dam body is not considered, but the lifting force and wetting deformation of the upstream rockfill in the reservoir water level change area are considered.

### 4.4. Simulation Results

According to the simulation process discussed above, the collapse settlement of the dams and the difference between the results of two different wetting deformation simulation methods are analyzed.

**Figure 10:**
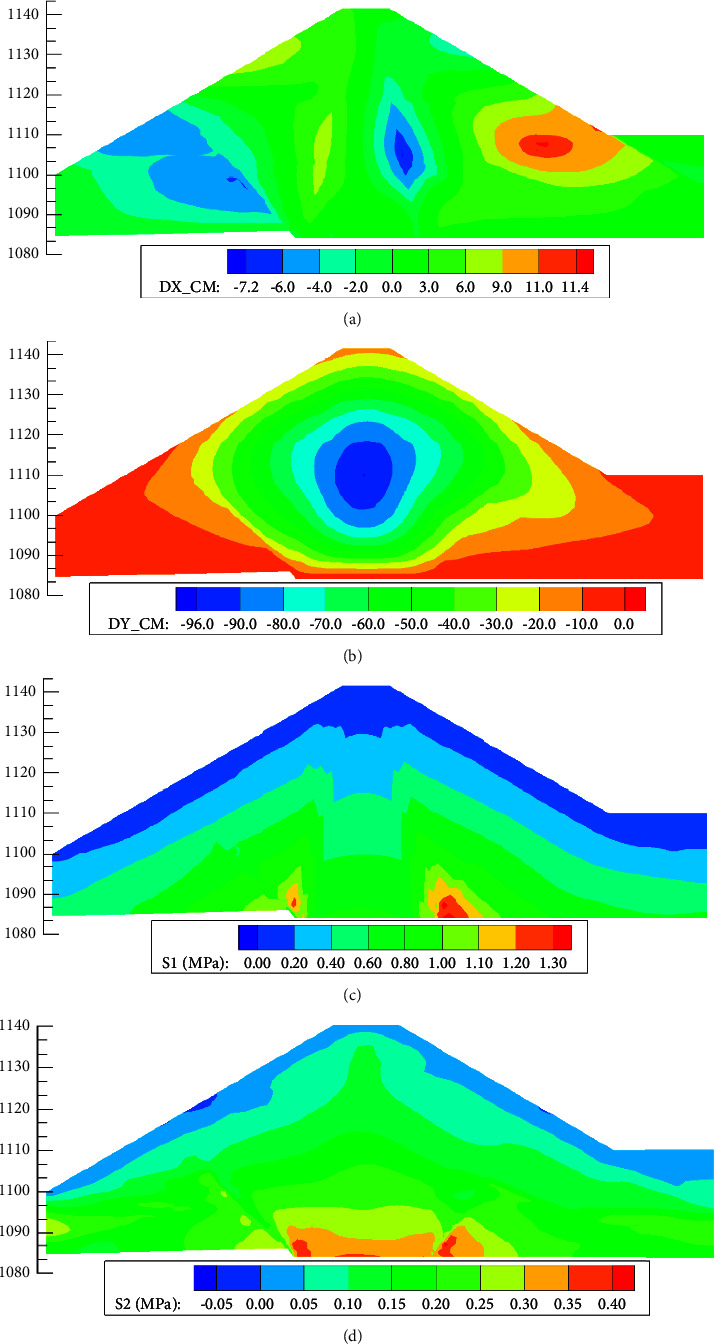
The displacement and stress contour maps at the completion period. (a) Horizontal displacement (cm). (b) Settlement (cm). (c) The major principal stress (cm). (d) The minor principal stress (cm).

Figure 10 shows the displacement nephogram and stress nephogram of the dam body during the completion period. Because the upstream cofferdam is filled first, the section size of the cofferdam is large, so the horizontal displacement of the dam body has no obvious symmetry. The maximum settlement of the dam body occurs at 1/2∼2/3 of the dam height, about −96.0 cm, and the ratio of the maximum settlement of the dam body to the dam height is 1.28%, which accords with the general law of dam deformation. There is no obvious tensile stress zone on the dam top, and no crack is produced.

**Figure 11:**
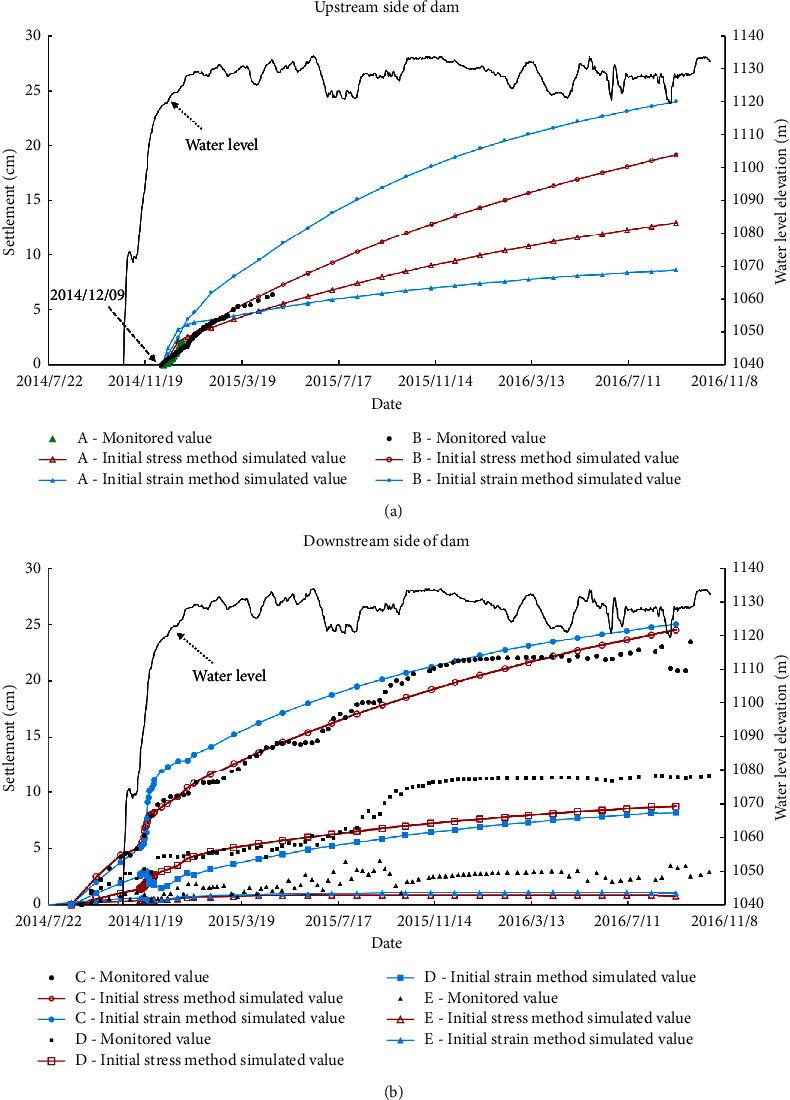
Comparison of monitoring values of settlement measuring points with simulated values of two methods. (a) Upstream side settlement. (b) Downstream side settlement.

Figure 11 shows the monitoring results of *A*–*E* (as shown in Figure 8) at the upstream dam slope, dam crest, and downstream dam slope of the dam and the comparison of the results simulated using the two methods. The marked points in the figure are the monitoring values, and the marked point-solid line is the finite element simulation value. It can be seen that the calculated settlement value of the dam is consistent with the actual measured value in distribution, and with the rise in the water level of the reservoir, the settlement amount increases during the impoundment. From the diagram, it can be seen that the wetting deformation mainly affects the deformation of the upstream dam slope (point *A*) and dam crest area (point *B* and point *C*) but has little influence on the deformation of the downstream dam slope (point *D* and point *E*).

Due to the influence of the water level change and other engineering factors, the monitoring data of measuring points *A* and *B* are very few, but it can still be seen from the figure that the results of the initial stress method simulation are close to the monitoring values. At point *C* on the crest of the dam, the simulation results of the initial stress method are well fitted with the monitoring values, whereas the simulation results of the initial strain method are larger. From August to November 2015, a large settlement mutation occurred at the measuring point *D* of the downstream dam slope. At this time, it was in the rainy season. It was considered that factors such as rainfall and downstream water level rise led to the wetting deformation of downstream rockfill, which was not considered in the simulation, so the fitting effect was poor. *E* point fitting is better. Therefore, the deformation simulated using the initial stress method is closer to the monitoring data, while the deformation simulated using the initial strain method is larger than the monitoring data, and the simulation results using the initial stress method can represent the actual deformation of this section during the impoundment period. Using the same parameters, the deformation simulated using the initial strain method is greater than that simulated by the initial stress method.

**Figure 12:**
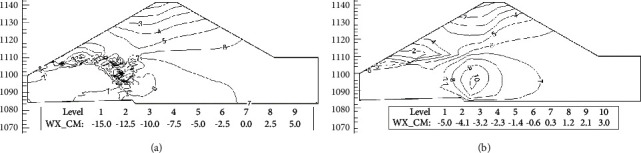
Horizontal displacement increment contour map (cm). (a) Initial strain method. (b) Initial stress method.

**Figure 13:**
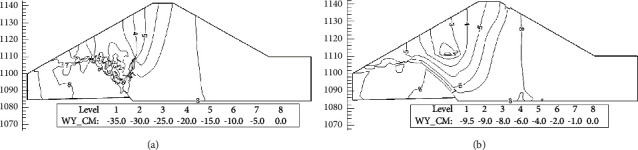
Settlement increment contour map (cm). (a) Initial strain method. (b) Initial stress method.

Figures 12 and 13 are the contour maps of horizontal displacement increment and settlement increment from before impoundment to water level reach 1111.0 m elevation. It can be seen from the diagram that the water storage makes the upper part of the dam produce obvious horizontal displacement to the upstream side, and the maximum horizontal displacement occurs on the upstream side of the dam crest. At the same time, due to the influence of wetting deformation, the upstream rockfill produced obvious collapse settlement and the maximum settlement occurred near the water level. The maximum displacement increment simulated using the initial strain method is about 3 times that of the initial stress method. At the same time, the displacement contour map simulated using the initial strain method is distributed in the area where the wetting deformation occurs, and there is a phenomenon of deformation inconsistency. Therefore, the initial stress method is recommended to simulate the wetting deformation.

**Figure 14:**
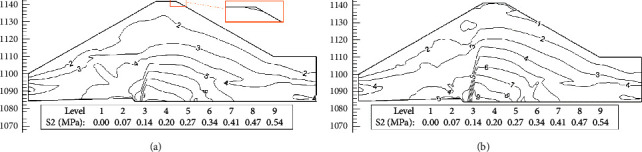
Contour of minor principal stress obtained by simulating wetting deformation with the initial stress method (MPa). (a) Water elevation 1111.0 m. (b) Water elevation 1120.0 m.

Figure 14 is the isoline of the minor principal stress of the section when the initial stress method is used to simulate the wetting deformation and water is stored at elevations of 1111.0 m and 1120.0 m. It can be seen from Figure 14 that when the water level reaches 1111.0 m, the dam crest appears in the tensile stress zone. With the rise in the water level, the tensile stress zone extends from the downstream side of the dam top to the downstream dam slope, then to the upstream side of the dam top, and finally to the upstream dam slope. In the area where the wetting deformation occurs, the stress distribution is relatively uniform, there is no stress singularity, and the tensile stress zone at the dam crest is consistent with the actual crack situation.

In addition, it can be seen from Figure 14 that as the water level rises, the wetting deformation of the upstream dam shell material causes the tensile stress zone to appear on the crest of the dam. Combined with the displacement increment diagram generated by the wetting effect in Figures 12 and 13, it can be seen that the wetting deformation is the direct cause of the cracks on the crest of the dam.

In the above simulation, the initial stress and initial strain are both loaded once and the calculation cost is the same; the parameters of the wetting model were obtained from indoor experiments. It can be seen from the simulation results that the simulation results of the initial stress method are more reasonable; therefore, the initial stress method should be preferred in the simulation of the wetting deformation of rockfill materials.

## 5. Conclusion

This paper introduces the *E*^*w*^ − *ν*^*w*^ model for calculating wetting deformation and its method for simulating wetting deformation. This model can not only calculate the strain vector of wetting deformation directly according to the generalized Hooke's law and simulate the wetting deformation of the core wall rockfill dam with the initial strain method but also deduce the stress reduction caused by wetting deformation according to the reduction of the secant modulus and simulate the wetting deformation with the initial stress method.

Using the same model and parameters, by comparing the simulation results of the initial strain method and the initial stress method, it is found that the simulated result using the initial stress method is in good agreement with the field monitoring data, the displacement simulated using the initial strain method is larger than that of the initial stress method, and the wetting deformation of the upstream rockfill material after impoundment is more serious. Moreover, the displacement contours simulated using the initial strain method are distributed in the area where the wetting deformation occurs, and there is a phenomenon of deformation inconsistency. It can be seen from the simulation results that the simulation results of the initial stress method are more reasonable; therefore, the initial stress method should be preferred in the simulation of the wetting deformation of rockfill materials.

At the same time, by simulating the filling and impoundment process of the 990.0 m cross section of Guanyinyan Dam, it is found that with the rise of the water level, the wetting deformation of the upstream dam shell material causes the tensile stress zone at the top of the dam, and the wetting deformation is the direct cause of the crack at the top of the dam.

## Figures and Tables

**Table 1:** E-B model parameters of main dam materials of the Guanyinyan rockfill dam.

Dam material	*γ* (10^4^ N/m^3^)	*K*	*n*	*R*_*f*_	*K*_*b*_	*m*	*ϕ*_0_ (°)	∆*ϕ* (°)	*c* (kPa)
Core wall	2.02	241	0.49	0.82	230	0.16	38.0	13.0	0.0
Filter I	2.08	400	0.30	0.70	200	0.22	43.5	7.5	0.0
Filter II	2.03	550	0.30	0.67	220	0.16	46.3	7.6	0.0
Rockfill I	2.15	583	0.27	0.66	280	0.14	48.6	8.1	0.0
Rockfill II	2.25	495	0.28	0.68	191	0.21	47.0	7.4	0.0

**Table 2:** Parameters of the rheological model of the Guanyinyan rockfill dam material.

Materials	Parameters						
	*b*_1_ (%)	*c*_1_ (%)	*d*_1_ (%)	*m*_1_	*m*_2_	*m*_3_	*α*
Core wall	1.700	0.031	0.100	0.600	0.900	0.302	0.0015
Rockfill	0.100	0.059	1.545	0.301	0.300	0.400	0.0051

**Table 3:** Parameters of the *E*^*w*^ − *ν*^*w*^ wetting model of rockfill.

Parameter	*K*_0_	*m*_0_	*K*_1_	*A*	*c*	*d*
Rockfill I	0.061	0.596	0.052	0.923	0.348	0.104
Rockfill II	0.070	0.606	0.052	1.027	0.349	0.110

## Data Availability

The data that support the findings of this study are included within the article [and/or its supplementary materials].
